# Hap-E Search 2.0: Improving the Performance of a Probabilistic Donor-Recipient Matching Algorithm Based on Haplotype Frequencies

**DOI:** 10.3389/fmed.2020.00032

**Published:** 2020-02-18

**Authors:** Christine Urban, Alexander H. Schmidt, Jan Andreas Hofmann

**Affiliations:** DKMS, Tübingen, Germany

**Keywords:** bioinformatics, HSCT, HLA, haplotype frequencies, matching algorithm

## Abstract

In the setting of hematopoietic stem cell transplantation, donor-patient HLA matching is the prime donor selection criterion. Matching algorithms provide ordered lists of donors where the probability of a donor to be an HLA match is calculated in cases where either donor or patient HLA typing information is ambiguous or incomplete. While providing important information for the selection of suitable donors, these algorithms are computationally demanding and often need several minutes up to hours to generate search results. Here, we present a new search kernel implementation for Hap-E Search, the haplotype frequency-based matching algorithm of DKMS. The updated search kernel uses pre-calculated information on donor genotypes to speed up the search process. The new algorithm reliably provides search results in <1 min for a large donor database (>9 Mio donors) including matching and mismatching donors, even for frequent or incomplete patient HLA data where the matching list contains several thousand donors. In these cases, the search process is accelerated by factors of 10 and more compared to the old Hap-E Search implementation. The predicted matching probabilities of the new algorithm were validated with data from verification typing requests of 67,550 donor-patient pairs.

## Introduction

Matching of donor and patient human leukocyte antigens (HLA) is a primary factor for patient outcome after hematopoietic stem cell transplantation (1–3). Over the years, newly registered potential stem cell donors have been typed with varying scope—from only two HLA loci (HLA-A and -B) to 5 or 6 loci [HLA-A, -B, -C, -DRB1, -DQB1 (and -DPB1)]—by applying different typing technologies from serology via SSO/SSP and Sanger sequencing to NGS. Therefore, the number of HLA loci typed and the resolution of typing results usually vary substantially between donors of the same registry.

In order to overcome this problem of missing or ambiguous data, several prediction tools—Haplo Stats at NMDP (4) and EasyMatch (5) at the French registry—as well as probabilistic matching algorithms—HapLogic at NMDP (6, 7), OptiMatch at ZKRD (8, 9), and Hap-E Search at DKMS (10)—have been developed. These matching algorithms use population-specific HLA haplotype frequencies (11–15) to determine the probability that an incompletely HLA-typed donor will be a 10/10 (9/10, 8/8, 7/8) match for a defined patient (considering HLA-A, -B, -C, -DRB1, -DQB1 for 10/10 and 9/10 matching and HLA-A, -B, -C, -DRB1 for 8/8 and 7/8 matching) when fully typed. In this way, important information for donor searches is provided.

WMDA has developed a framework for the implementation of HLA matching software (16) and carried out a reference study for the validation of such HLA matching algorithms (17). Hap-E Search, the search algorithm developed by DKMS in 2011, was one of seven matching algorithms participating in this study.

One drawback of standard matching algorithms is the poor runtime performance for large donor databases and haplotype datasets. It is desirable to accelerate the search process to make the donor selection workflow more efficient.

Here, we present the completely revised search kernel for Hap-E Search that meets the challenges of searching more than 9 million registered potential stem cell donors in an efficient way.

## Materials and Methods

### HF-Based Matching

Like HapLogic and OptiMatch, Hap-E Search uses standard formulae describing relations between haplotypes and genotypes in incompletely typed individuals to determine matching probabilities. Details are given in the Supplementary Material.

### Original Hap-E Search Algorithm

**Figure 1:**
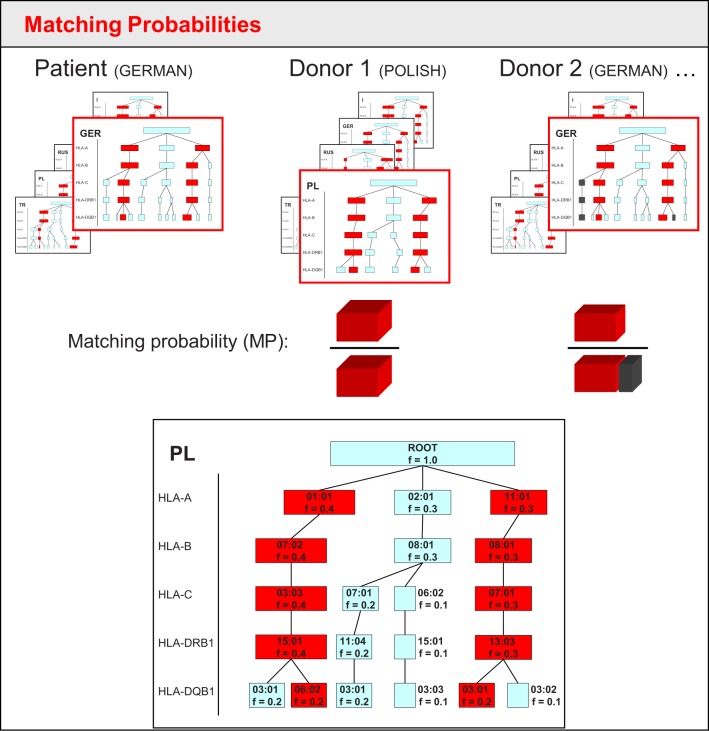
Sketch of matching probability calculation in the tree-based approach of the original Hap-E Search algorithm. Each frame schematically shows the tree-like structure in which haplotypes are stored for five different populations. The red frame indicates the corresponding haplotype tree matching the patient's/donor's ethnicity. For Donor 1, the simplified haplotype tree is shown in more detail below: each box is a node representing an allele of a haplotype locus. Each haplotype locus adds another level of nodes. The frequency *f* is stored for each node. To determine the matching frequency, the algorithm runs through all possibilities of haplotype pairs that lead to potential patient or donor genotypes. Red boxes within the tree indicate the matching haplotype pair for the genotype that patient and donor have in common. Gray boxes (as can be seen in the tree for donor 2) show haplotype pairs leading to potential donor genotypes that the patient does not share. Blue boxes represent haplotypes not associated with donor genotypes. As the patient in this example has high-resolution HLA typing, the matching probability is calculated by the frequency of the shared genotype (red) over the sum of all potential genotype frequencies of the donor (red for donor 1, red plus gray for donor 2).

Since 2011, the haplotype frequency-enhanced search algorithm Hap-E Search has been used at DKMS (10, 18). The first search kernel used a tree structure to store haplotype sets. This approach is numerically advantageous compared to full genotype lists because of the high degree of internal data structure. The haplotype tree has to be built only once for each haplotype dataset that is used for the haplotype matching. The former search kernel used haplotype data from German, Polish, Russian, Italian, and Turkish populations. For each population, a respective haplotype tree was built. The tree consists of nodes representing alleles. Each locus adds another level of nodes. The tree is built starting with the first locus of the haplotype (e.g., HLA-A). All distinct alleles of this locus are represented by a node. For each first-level node, second-level nodes are created by the haplotypes that share the same allele (i.e., first-level node) on the first locus. For these haplotypes, all different alleles on the second locus (e.g., HLA-B) are represented by second-level tree nodes linking to the first-level node. Repeating this procedure for all loci (HLA-A, -B, -C, -DRB1, and -DQB1) generates the whole tree. A single haplotype can then be seen as one path through the tree. During each search, the tree is used to determine genotypes of the patient and potentially matching donors. To determine genotype frequencies, it is necessary to run through all possible haplotype pairs that lead to the same genotype (Figure 1). Still, this method is more efficient in terms of computing time as well as required memory than a straightforward genotype-based approach without the use of a tree structure.

Nevertheless, with a continuously growing donor database and larger haplotype datasets, the tree-based approach is no longer sufficient to provide search results in adequate time, i.e., <1 min. Especially for donor searches that result in several thousand donors, search time increases substantially. With the new search kernel, we want to tune the performance of the search kernel and optimize user experience by providing search results in <1 min even for computationally demanding donor searches.

### Preparation of Donor Data

Our basic approach to improve the performance of Hap-E Search is to reduce the computational load during each donor search: the workload is transferred to a preparation step that has to be performed once before the search kernel gets operational. In this preparation step as much information as possible is stored and can then be re-used in every search, thus further reducing the time a user has to wait for search results. This approach is applied to potential donor genotypes based on HLA typing, as they are required in every search and change in rare cases only. In the preparation step before the new search kernel gets operational, we compute and store genotype information for all donors in the database. Afterwards, the donor information is constantly kept updated to the current state of the donor database.

**Figure 2:**
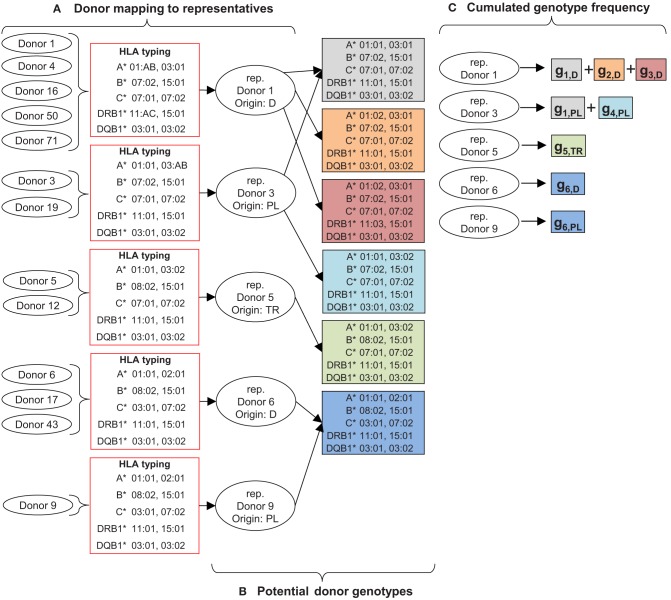
Sketch of donor data preparation for the new search kernel: **(A)** map all donors with the same HLA typing and ethnicity to one representative. **(B)** Determine potential donor genotypes according to HLA typing and haplotype data. **(C)** Calculate cumulated frequencies of all potential donor genotypes. The boxes represent the frequencies *g*_*i,e*_ of genotype *i* in the specified ethnicity *e*.

Currently, the search kernel uses 29,334 haplotypes from German, Polish, Russian, Italian, Turkish, and Indian populations for genotype assignment. Data preparation is illustrated in Figure 2. All donors with the same HLA typing and ethnicity are mapped to one representative to reduce the amount of donor data processed during the donor search (Figure 2A). For efficient and easy mapping, only identical typing results in terms of HLA nomenclature are grouped together, e.g., the two HLA typing results A^*^01:01 and A^*^01:01:01G would be assigned to different groups even though they are identical on antigen recognition domain (ARD) level. For our database, this grouping reduces the number of donors in the search kernel by 36%. More sophisticated grouping on ARD level was not implemented, as it improves the grouping effect only slightly (donor number reduction by 40% for our database) at much higher computational cost. For each representative, potential genotypes according to HLA typing results and underlying haplotype data are calculated (Figure 2B). Furthermore, the frequencies of all potential genotypes of a representative donor are summed up and stored (Figure 2C). This cumulated frequency is needed as denominator in the calculation of matching probabilities as described in the Supplementary Material.

For donors with incomplete HLA data, the stored genotype information is reduced to the loci with typing information. For example, when a donor was typed only for the loci HLA-A and -B, potential genotypes are summarized to “AB genotypes.”

The algorithm is programmed in a flexible way, so that population haplotype data can easily be expanded or added.

### Search Process

**Figure 3:**
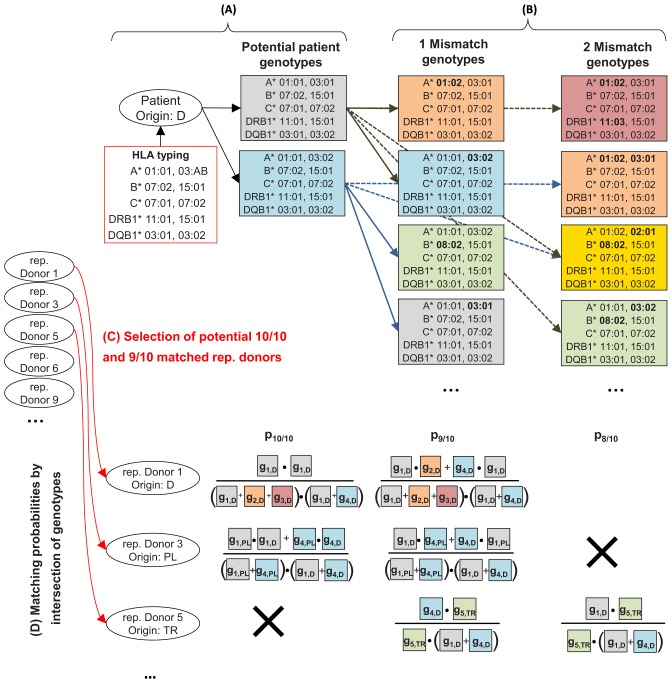
Sketch of the search process for exemplary patient typing: **(A)** Determine potential patient genotypes based on HLA typing. **(B)** Determine genotypes having one or two mismatches to the patient genotypes. **(C)** Select potential 10/10 and 9/10 matched representative donors (taken from Figure 2) based on HLA typing on antigen recognition domain level (independent of used haplotype data). **(D)** Calculate 10/10, 9/10, and 8/10 matching probabilities for all donor-patient pairs selected in (C) by intersecting patient and donor genotype information. *g*_*i,e*_ is the frequency of genotype *i* (color coded) in the population of ethnicity *e*. Match frequencies are calculated as described in the Supplementary Material. The numerator contains all genotypes that donor and patient have in common. The denominator multiplies the sums over the frequencies of all potential genotypes of donor and patient.

Donor-patient matching consists of several steps as outlined in Figure 3:

First, potential patient genotypes based on given patient HLA typing are determined (Figure 3A). Additionally, all genotypes having one or two mismatches to these patient genotypes are calculated (Figure 3B).

Then, all potential 10/10 and 9/10 matched representative donors are selected based on ARD matching (Figure 3C). This step is independent of used haplotype data in order to also select donors whose HLA typing cannot be represented by the given haplotypes.

10/10 and 9/10 (9/10 and 8/10) matching probabilities are calculated for all 10/10 (9/10) matched representative donors. The matching probabilities can be calculated efficiently (see Supplementary Material) by intersecting the 0/1/2 mismatch patient genotypes with pre-calculated donor genotype information (Figure 3D).

In a last step, results for representative donors are expanded to the full donor set.

For donors or patients with HLA typing that cannot be represented by the used haplotype data, we approximate matching probabilities by assuming minimal frequencies for the “unknown” genotypes. The minimal frequency is dependent on the sample size used for calculation of population haplotype data. For ambiguous typing results, all possible “unknown” genotypes are weighted equally. Details are given in the Supplementary Material.

### Expression-Level Characters and Homozygosity

There is an ongoing discussion whether HLA mismatches of loci that are homozygous for both patient and donor should be counted either as one or two mismatches. Therefore, this parameter can be changed in our search algorithm via a control variable. The default is one mismatch as specified in WMDA guidelines (16).

Table 1 gives an overview of the mismatch counting in the presence of null alleles for different HLA typing situations and examples. According to WMDA guidelines, alleles with expression-level suffices N, S, and C are treated as absent, i.e., the other HLA typing result of the locus is regarded to be homozygous (Table 1, C). However, while the homozygous typing result is used to find matching donors, matching probabilities are calculated with the haplotype frequency of the original null allele typing (not with the homozygous typing result).

**Table 1:** Overview of mismatch counting for HLA typing with homozygous loci or null alleles.

**Example**	**Patient typing**	**Donor typing**	**Mismatch count**	**Explanation**
A.	A^*^01:01, A^*^01:01	A^*^02:01, A^*^02:01	1^*^	Mismatches of loci that are homozygous for both patient and donor are counted as one mismatch.
B.	A^*^01:04N, A^*^34:01:01G	A^*^01:01:01G, A^*^34:01:01G	0	A^*^01:04N is member of the G-Group A^*^01:01:01G.
C.	A^*^01:04N, A^*^34:01:01G	A^*^34:01:01G, A^*^34:01:01G	0	Second patient allele is treated as homozygous.
D.	A^*^03:01, A^*^03:01	A^*^03:01, A^*^02:MRVF	0	Code A^*^02:MRVF contains null allele A^*^02:125N which is frequent enough to be represented in our haplotype data. This null portion of the typing is treated as homozygous and therefore as potential match.
E.	A^*^01:01, A^*^01:01	A^*^01:01, A^*^03:XX	0	Code A^*^03:XX contains null allele A^*^03:69N which is frequent enough to be represented in our haplotype data. This null portion of the typing is treated as homozygous and therefore as a potential match.
F.	A^*^03:01, A^*^03:01	A^*^03:01, A^*^01:01:01G	1	Even though the G-Code A^*^01:01:01G contains null alleles, these are rare and not represented in our haplotype data. The null portion of the typing is neglected in the mismatch counting.
G.	A^*^01:01, A^*^03:XX	A^*^01:01, A^*^02:MRVF	1	For a potential match of patient and donor, the null portion of both codes A^*^03:XX and A^*^02:MRVF would have to be considered. This unlikely scenario is ignored.
H.	A^*^01:04N, A^*^03:69N	A^*^34:01:01G, A^*^34:01:01G	1^*^	Locus with two null alleles is treated as homozygous but has no match with 34:01:01G.
I.	A^*^01:04N, A^*^03:69N	A^*^01:01:01G, A^*^01:01:01G	0	Locus with two null alleles is treated as homozygous. A^*^01:04N is member of the G-Group A^*^01:01:01.
J.	A^*^01:04N, A^*^03:69N	A^*^02:01, A^*^34:01:01G	2	Locus with two null alleles is treated as homozygous. The heterozygous typing of the donor leads to 2 mismatches.

* *In this overview, mismatches between homozygous loci are counted as single mismatches, as specified in the WMDA guidelines. The counting can be changed to two by adjusting a control variable in the algorithm*.

Additionally, null alleles can still match according to their genotype sequence: the high-resolution HLA typing result A^*^01:04N, A^*^34:01:01G matches A^*^01:01:01G, A^*^34:01:01G, as A^*^01:04N is part of the G-code A^*^01:01:01G (Table 1, B).

Some ambiguous HLA typing results potentially contain null alleles. For instance, the multi-allele code HLA-A^*^02:MRVF is composed of the HLA-A alleles 02:01, 02:70, 02:92, 02:113N, 02:125N, 02:217, 02:239, 02:249, and 02:260. If a null allele in the code is frequent enough to be represented in our haplotype data (in the example above: A^*^02:125N), this “null portion” of the multi-allele code is treated as homozygous for matching. This means that in addition to the standard matching of expressed alleles, the typing A^*^03:01+A^*^02:MRVF will also match like homozygous A^*^03:01+A^*^03:01 through its null portion A^*^03:01+A^*^02:125N (Table 1, D).

The algorithm can also handle two null alleles at the same locus. This locus with double-null typing is considered as homozygous and can only match with another potential double-null typing result (Table 1, I and J).

### Performance Tests: Setup

#### Comparison With the Original Hap-E Search Kernel

We compared the performance of the new search kernel to the original Hap-E Search kernel by evaluating the run time of 50 searches with high resolution patient typing that were randomly chosen from our productive system for both kernels. The searches were performed on our standard test environment—where the old search kernel could be used without adaptions—with 7.6 million donors using 27,651 haplotypes from German, Turkish, Russian, Italian, and Polish populations.

#### Performance in Different Settings

To further evaluate the efficiency of the new search kernel for different numbers of donors and haplotypes, we compared data preparation and search performance in three different controlled settings: 5 million donors and 12,407 haplotypes (setting 1), 1 million donors and 12,407 haplotypes (setting 2), and 1 million donors and 34,071 haplotypes (setting 3). All haplotypes in these settings were from the German population.

All tests were executed on an Oracle Database (Oracle Version 11.2) hosted on an Intel Xeon E5620 CPU (16 GB RAM, 6 cores on virtual machine, operating system SUSE Linux Enterprise Server 11).

On our productive system, searches can be processed in parallel on five dedicated oracle databases.

### Validation of Predicted Matching Probabilities: Setup

The new search kernel was validated both on simulated data provided by WMDA and on real donor-patient data from verification typing (VT) at our donor center, which is the patient-driven high-resolution retyping of donors upfront of an HSCT.

#### WMDA Matching Validation

WMDA has developed a cross-validation set for the validation of haplotype based search algorithms (17). It consists of data for 1,000 patients and 10,000 donors with varying typing profiles that was simulated based on 3,394 predefined haplotypes. WMDA provides a consensus dataset that contains the matching probabilities for all 10,000,000 donor-patient pairs. New search algorithms can be validated by running searches for this WMDA dataset and comparing the resulting matching probabilities to the consensus data.

#### Data From Verification Typing

Match predictions of the new search kernel were validated by comparing probability forecasts and actual match status of 67,550 donor-patient pairs from VT requests. Only donor-patient pairs where high resolution typing at all five match loci was available for both patient and donor after VT were used for validation. Donor typing at time of request and after VT were required to be consistent, such as to exclude typing errors from the validation samples.

10/10 and 9/10 matching probabilities were calculated with the new Hap-E Search kernel using the donor typing at time of VT request and German haplotype frequencies. Matching probabilities were rounded to integer percentage values and grouped into 22 classes in probability interval steps of 5%. Samples with a predicted 10/10 (9/10) probability of 0 or 100% were grouped into separate classes, as they account for most of the cases. The portion of VT confirmed matches within each class was then compared to the predicted value. For evaluation of the 10/10 matching probabilities, samples were excluded where the donor already had a confirmed mismatch to his patient prior to VT. This left 54,065 donor-patient pairs for validation in the 10/10 match category.

## Results

### Data Preparation

Preparation of donor data has to be performed once for the existing donor database before launch of the new search kernel. We compared data preparation for three different settings (Table 2). In both donor datasets, the proportion between different typing profiles was the same: 77% of the donors were typed for the loci HLA-A, -B, -C, -DRB1, and -DQB1; 10% were typed for HLA-A, -B, -C, and -DRB1; 9% were typed for HLA-A, -B, and -DRB1, and 3% had low-resolution typing of HLA-A and -B. Less than 1% had other typing profiles.

**Table 2:** Time required for the preparation of donor genotypes depending on the numbers of haplotypes and donors in the database.

**Duration of donor genotype preparation**				**Relative prep. duration**	
**Number of haplotypes**	**12,407**		**34,071**	**34,071/ 12,407**	**12,407**
**Number of donors**	**5 million**	**1 million**	**1 million**	**1 million**	**5 million/1 million**
**Setting**	**1**	**2**	**3**	**3 vs. 2**	**1 vs. 2**
Preparation for existing donor database	28 h 53 min	6 h 39 min	15 h 35 min	2.3	4.3
Preparation update for new donors	0.19 s	0.18 s	0.19 s	1.06	1.06
Average duration per day to keep up-to-date^*^	8 min 41 s	8 min 13 s	8 min 41 s	1.06	1.06

* *Assuming 1,000,000 new donors per year (on average 2,740 per day). The preparation was performed for three different settings (5 million donors and 12,407 haplotypes, 1 million donors and 12,407 haplotypes, 1 million donors and 34,071 haplotypes). For both 1 and 5 million donors, the proportion of different typing profiles was identical. The last two columns show the increase in preparation duration upon increase of haplotype and donor numbers*.

Depending on the setting, the time required for initial preparation ranged from 6 h 39 min to 28 h 53 min. For the settings tested, preparation time scales approximately proportional with size of donor database and number of haplotypes: For five times as many donors, preparation time increases by a factor of 4.3, while a 2.7-fold extension of haplotypes leads to an increase in preparation time by a factor of 2.3.

After launch of the search kernel, the genotype data must be kept updated permanently. In all three settings, about the same time (≈200 ms) is needed per updated or added donor for this process. Preparation time per donor is longer for the update step than in the initial preparation due to database administration overhead like table constraints, indices, etc.

Assuming a total of 1,000,000 new or updated donors per year (on average 2,740 per day), the cost of genotype data update would be <9 min per day in all three settings.

### Performance Tests: Results

#### Comparison With the Original Hap-E Search Kernel

We compared the performance of the new vs. the old search kernel for 50 searches as described in section comparison with the original Hap-E Search kernel. A direct comparison is difficult, as the two algorithms do not produce the same extent of search results: the new search kernel provides matching probabilities for all potential 9/10 and 10/10 matching donors, whereas the old search kernel excludes potential 9/10 donors where typing information is only available for loci HLA-A and HLA-B due to runtime issues. Therefore, the number of search results is considerably higher for the new compared to the old search kernel.

**Figure 4:**
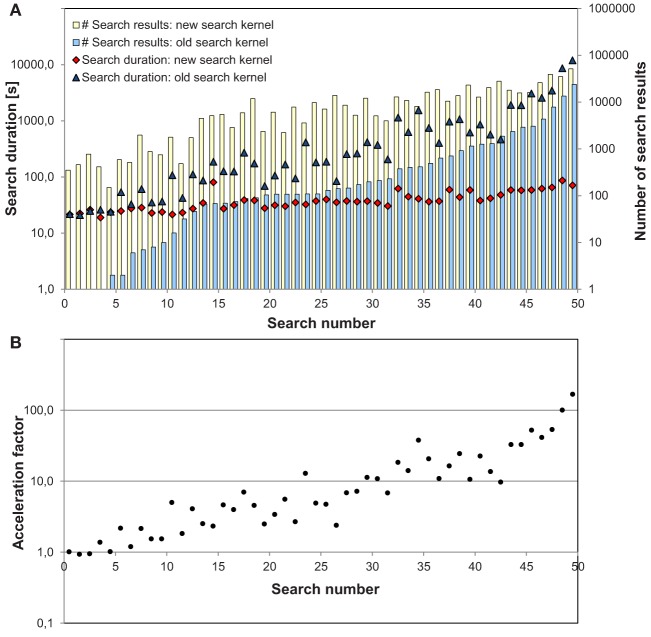
Comparison of the search duration with old and new search kernel for 50 searches: **(A)** for each search, bars indicate the number of search results for the new and the old search kernel. The old search excludes potential 9/10 donors where only typing on loci HLA-A and HLA-B is available. The search duration in seconds is shown for the old and the new search kernel. **(B)** Black dots show the factor by which searches are accelerated with the new search kernel compared to the old one for each search. Note that all values are shown on a logarithmic scale.

Figure 4 shows the results for all 50 searches. For searches that result in less then 10 donors with the old search kernel and about 100 donors with the new one, both kernels have similar search durations of 20–30 s. With growing number of search results, search duration increases steeply with the old search kernel, but only mildly with the new one—even though the number of search results is even higher. Search 50 with the highest number of search results (more than 50,000 for the new search kernel and almost 24,000 for the old one) finished in 70 s with the new search kernel. The old search kernel needed 167 times longer.

Compared to the former search kernel that used a tree-based approach, the match process is speeded up considerably (Figure 4B). The more expensive the search, the higher is the performance gain, especially for searches with more than 1,000 potential matches, where the search is accelerated by factors of 10 up to about 160.

#### Performance in Different Settings

We compared the average search duration for 15 arbitrary patients with 5-locus high-resolution HLA typing in the three settings described in section performance in different settings (Table 3). All searches were executed in <1 min.

**Table 3:** Average search duration depending on numbers of haplotypes and donors in the database.

**Search duration in seconds**				**Relative search duration**	
**Number of haplotypes**	**12,407**		**34,071**	**34,071/12,407**	**12,407**
**Number of donors**	**5 million**	**1 million**	**1 million**	**1 million**	**5 million/1 million**
**Setting**	**1**	**2**	**3**	**3 vs. 2**	**1 vs. 2**
Complete search	29.2 ± 12.2	14.4 ± 4.5	22.0 ± 9.6	1.5 ± 0.4	2.0 ± 0.5
Patient match and mismatch genotypes (A) & (B)	1.4 ± 0.6	1.4 ± 0.6	2.2 ± 1.2	1.7 ± 0.8	1.1 ± 0.5
Donor selection (C)	13.7 ± 5.7	9.2 ± 3.5	10.3 ± 5.4	1.1 ± 0.5	1.5 ± 0.4
Calculation of probabilities (D)	13.7 ± 9.7	3.6 ± 2.6	9.3 ± 8.2	2.5 ± 1.1	3.9 ± 1.2

*For 15 arbitrary patients with 5-locus high-resolution HLA typing, a donor search was performed in three different settings (5 million donors and 12,407 haplotypes, 1 million donors and 12,407 haplotypes, 1 million donors and 34,071 haplotypes). For each setting, average duration and standard deviation was calculated for all steps of the search. The last two columns show the increase in search duration upon increase of haplotype and donor numbers*.

As expected, preparation of patient genotype information (Figures 3A,B) is independent of the number of donors, while selection of potential 10/10 and 9/10 matched donors (Figure 3C) is independent of the number of used haplotypes. The computational cost of the calculation of matching probabilities (Figure 3D), is most susceptible to an increase in donor and haplotype numbers. Furthermore, it depends on the number of matching donors found by the specific search, which explains the large standard deviation of the duration across different searches.

Total search duration scales favorably with size of the donor database and number of used haplotypes: on average, it is doubled when the search is performed for a five times larger donor database. Average search duration increases by 50% when haplotypes are extended by a factor of 2.7. This makes it feasible to apply our approach to large and growing donor databases, and to improve calculated matching probabilities by using larger haplotype datasets.

### Validation of Predicted Matching Probabilities: Results

#### WMDA Matching Validation

We performed the matching validation on the simulated WMDA data (section WMDA Matching Validation) to evaluate our new algorithm. Matching probabilities for all 10,000,000 donor-patient pairs were identical to the consensus data, except one. For the pair P000656–D009637 we observed a deviation of 1% in the 9/10 probability (87 instead of 88%) due to rounding, which is within tolerance of the validation criteria. Average search duration per patient within this predefined donor-haplotype setting was 4.3 s with a standard deviation of 1.5 s and a median of 3.9 s. Maximum search duration was 17.7 s, whereas the fastest search took 2.7 s.

#### Data From Verification Typing

**Figure 5:**
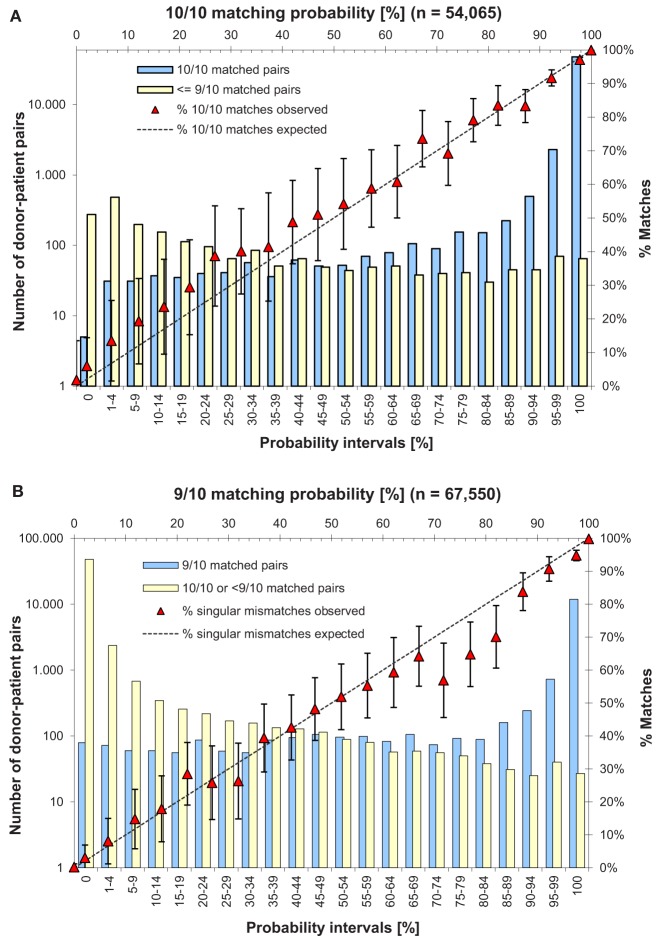
Comparison of probability forecasts and actual match status of donor-patient pairs from VT: **(A)** 10/10 matching category validated with 54,065 donor-patient pairs. **(B)** 9/10 matching category validated with 67,550 donor-patient pairs. Plot A (B) shows the observed portion of 10/10 (9/10) matches after VT over the expected value calculated with Hap-E Search in 22 probability interval classes. Error bars indicate the 95% confidence interval. Additionally, bars illustrate the number of donors who were confirmed to be a 10/10 (9/10) match and who were confirmed not to be a 10/10 (9/10) match by VT. The sum of both values is the total number of donor-patient pairs in this class.

The results for the validation of matching probabilities predicted by Hap-E Search on actual donor-patient data from VT is shown in Figure 5. The closer the observation points are to the intersecting line corresponding to the expected value, the more precise is our probability prediction.

For the 10/10 match category (Figure 5A), 93% of the 54,065 samples fall into the two highest probability classes (95–99 and 100%). For the 9/10 match category (Figure 5B), 89% of the 67,550 samples fall into the highest or lowest probability class (0 and 100%).

The data confirm the validity of our predicted matching probabilities: 21/22 data points are within 95% confidence interval for the 10/10 match category, and 17/22 data points for the 9/10 match category.

## Discussion

This work focuses on improving the software side of the donor selection process: we present a new approach that tuned the performance of our matching algorithm Hap-E Search for a more efficient workflow within standard donor center and registry activities at DKMS.

We tested data preparation and search performance for three different settings to estimate the behavior of the algorithm depending on the number of donors and haplotypes. Data preparation time scales approximately proportional to both parameters, which is beneficial especially for large donor databases. However, the duration of initial data preparation is of minor importance (as long as it does not deteriorate): it needs only to be performed once before the launch of the search kernel or after changes of the haplotype basis. This preparation can be performed in the background so that the search service does not need to be interrupted in the meantime.

More importantly, the time needed for the constant update of the database was negligible with <9 min per day (assuming 1,000,000 new or updated donors per year) in all settings.

The duration of donor searches scales favorably with the number of donors in the database as well as the number of used haplotypes, which makes it feasible to apply our approach to large and growing donor databases, and to improve calculated matching probabilities by using larger haplotype datasets. The algorithm is programmed in a flexible way, so that haplotype data can easily be extended to improve the accuracy of matching probabilities.

The new search kernel has been operational since September 2018 and provides fast and robust search results. Currently, the donor database contains over 9 million donors from six different DKMS entities in Germany, Poland, United Kingdom, United States, Chile and India. At the moment, we run over 3,000 new searches per month, of which ~500 are registry searches and 2,500 are searches connected to donor center activities. The majority of searches in the registry are triggered via the European Marrow Donor Information System (EMDIS) (19, 20). Ninety-five percent of the user searches during the day can be started without delay and do not have to wait for other searches to finish.

For all active searches, search results are updated on a daily basis to report changes in the matching list. At the moment, we run search updates for about 2,200 registry searches (mostly EMDIS) and 180 donor center searches each night. These search updates are processed within 4.5 h.

The performance improvement achieved by the new search kernel directly improves the day-to-day work of search coordinators, who are the main users of the Hap-E Search algorithm. The reduced waiting time until search results are available facilitates a smoother and more effective work flow.

With the improvements of Hap-E Search 2.0, we now have a fast, reliable, and flexible matching algorithm that meets the challenges of a large and further growing donor database.

## Data Availability Statement

The datasets generated for this study are available on request to the corresponding author.

## Author Contributions

CU and JH: development of the search algorithm. CU: manuscript. AS: supervision, advise, and sponsorship.

### Conflict of Interest

The authors declare that the research was conducted in the absence of any commercial or financial relationships that could be construed as a potential conflict of interest.
