# Supplementary material for: Hap-E Search 2.0: Improving the Performance of a Probabilistic Donor-Recipient Matching Algorithm Based on Haplotype Frequencies
Source: Front Med (Lausanne). 2020 Feb 18;7:32. doi: 10.3389/fmed.2020.00032 (PMC7040233; doi:10.3389/fmed.2020.00032)
Supplement: Supplementary file 1 [file Data_Sheet_1.PDF]

Supplementary Material

1 Haplotype frequency-based matching prediction

Population-specific HF can be used to calculate matching probabilities for donors with incomplete or ambiguous HLA typing. Here, we describe the standard formulae used by probabilistic matching algorithms like HapLogic, OptiMatch and Hap-E Search.

All potential genotypes that are consistent with the donor typing have to be inferred from the haplotype dataset for a given donor ethnicity. As genotypes obtained with standard sequencing methods in the stem cell donor registry context contain no phase information, the same genotype can be obtained by combining different pairs of haplotypes. The frequency of genotype i for ethnicity e, $g_i(e)$, is the sum over the product of haplotype frequencies of all haplotype pairs $h_l h_k$ that lead to the same genotype g_i,

$$g_i(e) = \sum_{h_l h_k \in g_i} h_l h_k$$

The probability that genotype i is existent in the donor is then

$$p_i = \frac{g_i(e_{Donor})}{\sum_{k \in DonorGenotypes} g_k(e_{Donor})} = \frac{g_i(e_{Donor})}{g_{all,Donor}(e_{Donor})}$$

where $g_{all,Donor}(e_{Donor})$ is the sum over the frequency of all potential donor genotypes in the donor's ethnicity e_{Donor}. Potential genotypes and genotype frequencies of the patient are obtained in an analogous manner.

The probability that a donor is a 10/10 match for a patient is the probability that donor and patient share the same genotype, which is calculated as

$$p_{match} = \frac{1}{g_{all,Donor} \cdot g_{all,Patient}} \sum_{\substack{i \in \\ Donor\ Genotypes \\ \cap \\ Patient\ Genotypes}} [g_i(e_{Donor}) \cdot g_i(e_{Patient})]$$

The sum runs over all potential genotypes that patient and donor have in common. $g_{all,Patient}(e_{Patient})$ is the cumulated frequency of potential patient genotypes in the patient's ethnicity $e_{Patient}$. In the case of high resolution patient typing the formula simplifies to

$$p_{match} = \frac{\delta \cdot g_{Patient}(e_{Donor})}{g_{all,Donor}}$$

with $g_{Patient}(e_{Donor})$ being the frequency of the (unambiguous) patient genotype in the donor's ethnicity. δ takes the value 1 if the patient genotype is also a potential donor genotype and 0 otherwise.

To calculate the probability that a donor is a 9/10 match for a patient with mismatch on locus L, all potential donor genotypes that have the specified mismatch to any potential patient genotype have to be considered:

$$p_{1MM,locus\ L} = \frac{1}{g_{all,Donor} \cdot g_{all,Patient}} \sum_{\substack{j \in \\ Patient\ Genotypes}} g_j(e_{Patient}) \sum_{\substack{i \in \\ Donor\ Genotypes \\ with\ 1MM\ on\ locus\ L \\ compared\ with \\ Patient\ Genotype\ j}} g_i(e_{Donor})$$

The overall 9/10 probability is

$$p_{1MM} = \sum_{L \in \{A,B,C,DRB1,DQB1\}} p_{1MM,L}$$

The probability that a donor is an 8/10 match for a patient is calculated similarly, considering all potential donor genotypes that have two mismatches to any potential patient genotype. The two mismatches can lie on the same locus or on two different loci.

$$p_{2MM,loci\ L1L2} = \frac{1}{g_{all,Donor} \cdot g_{all,Patient}} \sum_{\substack{j \in \\ Patient\ Genotypes}} g_j(e_{Patient}) \sum_{\substack{i \in \\ Donor\ Genotypes \\ with\ first\ MM\ on\ locus\ L1 \\ and\ second\ MM\ on\ locus\ L2 \\ compared\ with \\ Patient\ Genotype\ j}} g_i(e_{Donor})$$

The overall 8/10 probability is

$$p_{2MM} = \sum_{\substack{L1 \in \{A,B,C,DRB1,DQB1\} \\ L2 \in \{A,B,C,DRB1,DQB1\}}} p_{2MM,L1L2}$$

.

2 Matching prediction for donors with unknown genotypes

For donors or patients with HLA typing results that cannot be represented by the haplotype data used, we approximate matching probabilities by assuming minimal frequencies for the "unknown" genotypes. The minimal frequency is dependent on the sample size N_e used for calculation of haplotype data for population e, $g_{min}(e) = \frac{1}{(2N_e)^2}$. For ambiguous typing results, all possible "unknown" genotypes are weighted equally. The sum over all potential donor genotype frequencies in the case of ambiguous typing and unknown genotype is

$$g_{all,Donor} = n_{Genos} g_{min}(e_{Donor})$$

where n_{Genos} is the number of potential genotypes that can be constructed with ARD combinations based on donor typing.
